# Tetra­methyl *N*,*N*′-(2,2,3,3,4,4-hexa­fluoro-1,5-dioxopentane-1,5-di­yl)bis­(phospho­ramidate)

**DOI:** 10.1107/S1600536812011191

**Published:** 2012-03-21

**Authors:** Victor A. Trush, Kateryna E. Gubina, Yaroslav O. Gumeniuk, Tetyana Yu. Sliva, Irina S. Konovalova

**Affiliations:** a National Taras Shevchenko University, Department of Chemistry, Volodymyrska str. 64, 01601 Kyiv, Ukraine; b National University of Life and Environmental Sciences of Ukraine, Heroiv Oboroni str. 15, 03041 Kyiv, Ukraine; c STC "Institute for Single Crystals", 60 Lenina ave., Khar’kov 61001, Ukraine

## Abstract

The mol­ecule of the title compound, C_9_H_14_F_6_N_2_O_8_P_2_, lies on a twofold rotation axis that passes through the middle C atom of the three-atom fluoro­methyl­ene unit. The carbonyl and phosphoryl groups are in an antiperiplanar conformation. In the crystal, N—H⋯O=P hydrogen bonds link the mol­ecules into polymeric chains parallel to the *c* axis.

## Related literature

For background to the chemistry of phospho­rus–organic compounds, see: Ly & Woollins (1998 ▶). For the biological and pharmacological properties of carbacyl­amido­phosphate der­iv­atives, see: Adams *et al.* (2002 ▶). For details of the synthesis and properties of phospho­ramide derivatives, see: Kirsanov & Levchenko (1957 ▶); For structural analogues of phospho­rylated carbacyl­amides and their properties, see: Trush *et al.* (2005 ▶); Gubina *et al.* (2000 ▶). For the synthesis and properties of fluorinated compounds, see: Leontieva *et al.* (2002 ▶).
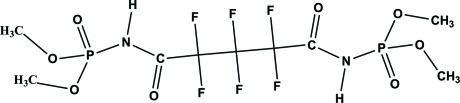


## Experimental

### 

#### Crystal data

C_9_H_14_F_6_N_2_O_8_P_2_
*M* *_r_* = 454.16
Monoclinic, 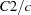

*a* = 19.7862 (13) Å
*b* = 5.2801 (4) Å
*c* = 16.9943 (11) Å
β = 100.427 (6)°
*V* = 1746.1 (2) Å^3^
*Z* = 4
Mo *K*α radiation
μ = 0.35 mm^−1^
*T* = 293 K
0.40 × 0.20 × 0.10 mm

#### Data collection

Oxford Diffraction Xcalibur3 diffractometer
Absorption correction: multi-scan (*CrysAlis PRO*; Oxford Diffraction, 2010 ▶) *T*_min_ = 0.872, *T*_max_ = 0.966
7084 measured reflections
2504 independent reflections
1669 reflections with *I* > 2σ(*I*)
*R*_int_ = 0.023
2 standard reflections every 50 reflections intensity decay: 0.3%

#### Refinement

*R*[*F* ^2^ > 2σ(*F* ^2^)] = 0.037
*wR*(*F* ^2^) = 0.106
*S* = 0.93
2504 reflections
125 parameters
H-atom parameters constrained
Δρ_max_ = 0.29 e Å^−3^
Δρ_min_ = −0.24 e Å^−3^

### 

Data collection: *CrysAlis CCD* (Oxford Diffraction, 2006 ▶); cell refinement: *CrysAlis CCD*; data reduction: *CrysAlis RED* (Oxford Diffraction, 2006 ▶); program(s) used to solve structure: *SHELXS97* (Sheldrick, 2008 ▶); program(s) used to refine structure: *SHELXL97* (Sheldrick, 2008 ▶); molecular graphics: *XP* in *SHELXTL* (Sheldrick, 2008 ▶); software used to prepare material for publication: *PLATON* (Spek, 2009 ▶).

## Supplementary Material

Crystal structure: contains datablock(s) I, global. DOI: 10.1107/S1600536812011191/fj2523sup1.cif

Structure factors: contains datablock(s) I. DOI: 10.1107/S1600536812011191/fj2523Isup2.hkl

Supplementary material file. DOI: 10.1107/S1600536812011191/fj2523Isup3.cml

Additional supplementary materials:  crystallographic information; 3D view; checkCIF report

## Figures and Tables

**Table 1:** Hydrogen-bond geometry (Å, °)

*D*—H⋯*A*	*D*—H	H⋯*A*	*D*⋯*A*	*D*—H⋯*A*
N1—H1*NA*⋯O4^i^	0.86	1.93	2.7750 (17)	168

Symmetry code: (i) 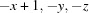.

**Fig. 1.**
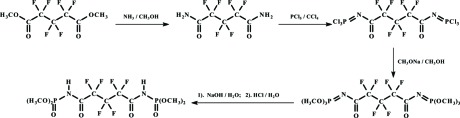
Scheme of synthesis of tetramethyl (2,2,3,3,4,4-hexafluoro-1,5-dioxopentane-1,5-diyl)bis(amidophosphate).

**Fig. 2.**
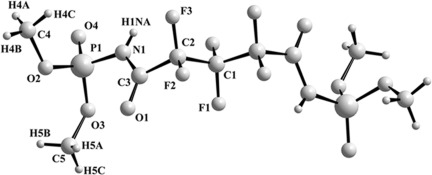
A view of tetramethyl(2,2,3,3,4,4-hexafluoro-1,5-dioxopentane-1,5-diyl)bis(amidophosphate). Symmetry transformation used to generate equivalent atoms: i – x + 1,y,-z + 1/2.

**Fig. 3.**
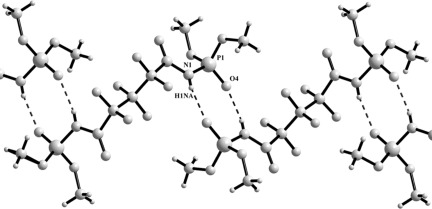
A system of hydrogen bonds as a one-dimensional polymeric ladder chains.

## supplementary crystallographic information

### Comment

Tetramethyl (2,2,3,3,4,4-hexafluoro-1,5-dioxopentane-1,5-diyl)bis(amidophosphate) is a representative of the carbacylamidophospates (CAPh), a family of compounds containing C(O)NHP(O) group. The presence of a peptide group in the carbacylamidophospates causes its diverse biological activity. (Adams *et al.*, 2002). CAPh may be regarded as powerful chelating ligand systems. There has recently been a resurgence of interest in their coordination chemistry as a consequence of the steric control that this ligand system may impart compared to, for example, _β_-diketonates. The wide range of coordination compounds were synthesized and described in detailes (Ly & Woollins, 1998).

The crystal structure of tetramethyl (2,2,3,3,4,4-hexafluoro-1,5-dioxopentane-1,5-diyl)bis(amidophosphate) (1) reveals, that the molecule of phosphorylated amide consists of symmetric moiety lying on a twofold rotation axis that passes through the middle atom of the three-atom fluoromethylene unit (- *x*,*y*,-*z* + 1/2). CF_2_– groups are situated in the retarded conformation to each other with the values of dihedral FCCF angles in the range 57.76 - 68.62° (Fig.2). In the molecule, the carbonyl and phosphoryl groups are in antiperiplanar conformation. The frame O4—P1—N1—C3—O1 is almost flat (the values of dihedral O4—P1—N1—C3 and O1—C3—N1—P1 angles are -169.54 and -0.05°, respectively) as it has been observed for the most CAPh (Gubina *et al.*, 2000; Trush *et al.* 2005). The crystal is composed from polymer chains which are built from molecules linked *via* intermolecular hydrogen N—H···O=P bonds (Fig.3). The parameters of the intermolecular hydrogen bond are listed in Table 1.

### Experimental

The compound tetramethyl(2,2,3,3,4,4-hexafluoro-1,5-dioxopentane-1,5-diyl)bis(amidophosphate) (1) can be synthesized by multistep reaction (Fig.1) starting from dimethyl hexafluoropentanedioate (Leontieva *et al.* 2002). The solution of 26.81 g (0.1 mol) dimethyl hexafluoropentanedioate in 40 ml of methanol was added drop-wise to the well stirred saturated solution of ammonia in methanol (~ 200 ml) under cooling. The obtained mixture was allowed to stand for a weak. Then solvent was removed under reduced pressure to give the crude product. Recrystallization from water gave the white powder of 2,2,3,3,4,4-hexafluoropentanediamide (22.6 g, 95%). Subsequently, the meticulous dried diamide (11.91 g, 0.05 mol) was involved in phosphoroazo-reaction (Kirsanov & Levchenko, 1957) with 20.82 g (0.1 mol) of PCl_5_ in 10 ml CCl_4_. The treatment of crude hexachloroanhydride with NaOCH_3_ (0.3 mol) in methanol solution leads to obtain hexaester with good yield. Further alkaline hydrolysis and acidification gave the final product - tetramethyl(2,2,3,3,4,4-hexafluoro-1,5-dioxopentane-1,5-diyl)bis(amidophosphate) (yield 13.6 g, 60%). The single crystals of 1 suitable for X-ray analysis were grown from aqueous-methanol solution (1:1).

### Refinement

All H atoms were placed at calculated positions and treated as riding on their parent atoms [C—H = 0.96 Å, and *U*_iso_(H) = 1.5*U*_eq_(C), N—H = 0.86 Å and *U*_iso_(H) = 1.2*U*_eq_(N)].

### Crystal data

**Table:**

C_9_H_14_F_6_N_2_O_8_P_2_	*F*(000) = 920
*M**_r_* = 454.16	*D*_x_ = 1.728 Mg m^−^^3^
Monoclinic, *C*2/*c*	Mo *K*α radiation, λ = 0.71073 Å
Hall symbol: -C 2yc	Cell parameters from 2504 reflections
*a* = 19.7862 (13) Å	θ = 2.9–30°
*b* = 5.2801 (4) Å	µ = 0.35 mm^−^^1^
*c* = 16.9943 (11) Å	*T* = 293 K
β = 100.427 (6)°	Block, colourless
*V* = 1746.1 (2) Å^3^	0.40 × 0.20 × 0.10 mm
*Z* = 4	

### Data collection

**Table:**

Oxford Diffraction Xcalibur3 diffractometer	1669 reflections with *I* > 2σ(*I*)
Radiation source: fine-focus sealed tube	*R*_int_ = 0.023
Graphite monochromator	θ_max_ = 30.0°, θ_min_ = 2.9°
ω scans	*h* = −27→27
Absorption correction: multi-scan (*CrysAlis PRO*; Oxford Diffraction, 2010)	*k* = −7→7
*T*_min_ = 0.872, *T*_max_ = 0.966	*l* = −23→22
7084 measured reflections	2 standard reflections every 50 reflections
2504 independent reflections	intensity decay: 0.3%

### Refinement

**Table:**

Refinement on *F*^2^	Primary atom site location: structure-invariant direct methods
Least-squares matrix: full	Secondary atom site location: difference Fourier map
*R*[*F*^2^ > 2σ(*F*^2^)] = 0.037	Hydrogen site location: inferred from neighbouring sites
*wR*(*F*^2^) = 0.106	H-atom parameters constrained
*S* = 0.93	*w* = 1/[σ^2^(*F*_o_^2^) + (0.0667*P*)^2^] where *P* = (*F*_o_^2^ + 2*F*_c_^2^)/3
2504 reflections	(Δ/σ)_max_ = 0.001
125 parameters	Δρ_max_ = 0.29 e Å^−^^3^
0 restraints	Δρ_min_ = −0.24 e Å^−^^3^

### Special details

**Table:**

Experimental. Analysis found: IR (KBr pellet, cm^-1^): 3095(s, NH), 2925(ns, CH), 1190(*s*), 1746(s, C=O), 1478(s CN); 1291 (as, CF), 1212 (s, PO), 1141 (s, CF). NMR - ^1^H (DMSO-d^6^): C—H 3.74 (*d*) 12H, ^3^J_PH_ = 11.6 Hz; NH 11.38 (*d*) 2H; ^31^P (DMSO-d^6^): -0.28 (hept) ^3^J_HP_ = 11.6 Hz; ^13^C (DMSO-d^6^): C(O) 159.68, CF 108.6 - 105.9, CH_3_ 54.74.
Geometry. All s.u.'s (except the s.u. in the dihedral angle between two l.s. planes) are estimated using the full covariance matrix. The cell s.u.'s are taken into account individually in the estimation of s.u.'s in distances, angles and torsion angles; correlations between s.u.'s in cell parameters are only used when they are defined by crystal symmetry. An approximate (isotropic) treatment of cell s.u.'s is used for estimating s.u.'s involving l.s. planes.
Refinement. Refinement of *F*^2^ against ALL reflections. The weighted *R*-factor *wR* and goodness of fit *S* are based on *F*^2^, conventional *R*-factors *R* are based on *F*, with *F* set to zero for negative *F*^2^. The threshold expression of *F*^2^ > 2σ(*F*^2^) is used only for calculating *R*-factors(gt) *etc*. and is not relevant to the choice of reflections for refinement. *R*-factors based on *F*^2^ are statistically about twice as large as those based on *F*, and *R*- factors based on ALL data will be even larger.

### Fractional atomic coordinates and isotropic or equivalent isotropic displacement parameters (Å^2^)

**Table:**

	*x*	*y*	*z*	*U*_iso_*/*U*_eq_	
F1	0.54045 (6)	−0.1156 (2)	0.30276 (6)	0.0559 (3)	
F2	0.58399 (6)	−0.5823 (2)	0.25478 (7)	0.0568 (3)	
F3	0.50776 (6)	−0.5503 (2)	0.14516 (6)	0.0495 (3)	
P1	0.61282 (2)	0.06932 (8)	0.04812 (2)	0.03549 (13)	
N1	0.57021 (7)	−0.1298 (3)	0.09836 (8)	0.0368 (3)	
H1NA	0.5280	−0.1619	0.0784	0.044*	
O1	0.65572 (7)	−0.2202 (3)	0.20427 (8)	0.0509 (3)	
O2	0.67108 (6)	−0.0844 (2)	0.01929 (7)	0.0470 (3)	
O3	0.65216 (6)	0.2593 (2)	0.10883 (7)	0.0458 (3)	
O4	0.56309 (6)	0.1876 (3)	−0.01514 (7)	0.0463 (3)	
C1	0.5000	−0.2656 (4)	0.2500	0.0349 (5)	
C2	0.54710 (9)	−0.4170 (3)	0.20389 (9)	0.0368 (3)	
C3	0.59784 (8)	−0.2443 (3)	0.16861 (9)	0.0359 (3)	
C4	0.65304 (12)	−0.2569 (4)	−0.04796 (13)	0.0609 (6)	
H4C	0.6191	−0.3749	−0.0369	0.091*	
H4B	0.6933	−0.3475	−0.0561	0.091*	
H4A	0.6349	−0.1621	−0.0953	0.091*	
C5	0.72440 (10)	0.2612 (5)	0.14340 (16)	0.0672 (6)	
H5C	0.7340	0.4008	0.1799	0.101*	
H5B	0.7509	0.2787	0.1017	0.101*	
H5A	0.7363	0.1054	0.1716	0.101*	

### Atomic displacement parameters (Å^2^)

**Table:**

	*U*^11^	*U*^22^	*U*^33^	*U*^12^	*U*^13^	*U*^23^
F1	0.0650 (7)	0.0573 (7)	0.0483 (6)	−0.0249 (6)	0.0182 (6)	−0.0188 (5)
F2	0.0620 (7)	0.0545 (7)	0.0567 (7)	0.0208 (6)	0.0179 (6)	0.0233 (5)
F3	0.0586 (7)	0.0482 (6)	0.0447 (6)	−0.0125 (5)	0.0172 (5)	−0.0133 (5)
P1	0.0276 (2)	0.0451 (2)	0.0324 (2)	−0.00321 (17)	0.00186 (15)	0.00114 (16)
N1	0.0276 (7)	0.0485 (8)	0.0325 (6)	−0.0032 (6)	0.0008 (5)	0.0049 (6)
O1	0.0375 (7)	0.0623 (8)	0.0471 (7)	−0.0022 (6)	−0.0077 (6)	0.0082 (6)
O2	0.0322 (6)	0.0634 (8)	0.0463 (7)	−0.0026 (6)	0.0093 (5)	−0.0114 (6)
O3	0.0347 (6)	0.0518 (7)	0.0479 (7)	−0.0046 (5)	−0.0009 (5)	−0.0094 (5)
O4	0.0367 (6)	0.0573 (7)	0.0413 (7)	−0.0064 (6)	−0.0026 (5)	0.0145 (5)
C1	0.0389 (12)	0.0357 (11)	0.0296 (10)	0.000	0.0045 (9)	0.000
C2	0.0420 (9)	0.0365 (8)	0.0311 (7)	0.0032 (7)	0.0042 (6)	0.0042 (6)
C3	0.0337 (8)	0.0413 (8)	0.0317 (7)	0.0025 (7)	0.0032 (6)	0.0009 (6)
C4	0.0603 (13)	0.0614 (13)	0.0622 (13)	−0.0049 (11)	0.0143 (11)	−0.0208 (10)
C5	0.0381 (10)	0.0707 (14)	0.0843 (15)	−0.0082 (10)	−0.0121 (10)	−0.0189 (12)

### Geometric parameters (Å, º)

**Table:**

F1—C1	1.3461 (16)	O3—C5	1.444 (2)
F2—C2	1.3469 (18)	C1—F1^i^	1.3461 (16)
F3—C2	1.3475 (18)	C1—C2^i^	1.545 (2)
P1—O4	1.4598 (12)	C1—C2	1.545 (2)
P1—O3	1.5444 (12)	C2—C3	1.555 (2)
P1—O2	1.5595 (13)	C4—H4C	0.9600
P1—N1	1.6751 (14)	C4—H4B	0.9600
N1—C3	1.361 (2)	C4—H4A	0.9600
N1—H1NA	0.8600	C5—H5C	0.9600
O1—C3	1.202 (2)	C5—H5B	0.9600
O2—C4	1.454 (2)	C5—H5A	0.9600
			
O4—P1—O3	113.89 (8)	F3—C2—C1	108.98 (12)
O4—P1—O2	115.43 (8)	F2—C2—C3	108.38 (13)
O3—P1—O2	103.65 (7)	F3—C2—C3	110.33 (12)
O4—P1—N1	108.04 (7)	C1—C2—C3	112.52 (14)
O3—P1—N1	107.84 (7)	O1—C3—N1	126.17 (16)
O2—P1—N1	107.59 (7)	O1—C3—C2	119.38 (14)
C3—N1—P1	124.51 (11)	N1—C3—C2	114.45 (13)
C3—N1—H1NA	117.7	O2—C4—H4C	109.5
P1—N1—H1NA	117.7	O2—C4—H4B	109.5
C4—O2—P1	118.85 (13)	H4C—C4—H4B	109.5
C5—O3—P1	128.21 (14)	O2—C4—H4A	109.5
F1—C1—F1^i^	107.92 (19)	H4C—C4—H4A	109.5
F1—C1—C2^i^	107.89 (7)	H4B—C4—H4A	109.5
F1^i^—C1—C2^i^	107.56 (8)	O3—C5—H5C	109.5
F1—C1—C2	107.56 (8)	O3—C5—H5B	109.5
F1^i^—C1—C2	107.89 (7)	H5C—C5—H5B	109.5
C2^i^—C1—C2	117.67 (19)	O3—C5—H5A	109.5
F2—C2—F3	108.08 (13)	H5C—C5—H5A	109.5
F2—C2—C1	108.44 (12)	H5B—C5—H5A	109.5
			
O4—P1—N1—C3	−169.53 (14)	F1^i^—C1—C2—F3	−57.75 (17)
O3—P1—N1—C3	−46.00 (15)	C2^i^—C1—C2—F3	64.07 (10)
O2—P1—N1—C3	65.23 (15)	F1—C1—C2—C3	−51.23 (15)
O4—P1—O2—C4	−45.46 (17)	F1^i^—C1—C2—C3	64.95 (15)
O3—P1—O2—C4	−170.70 (14)	C2^i^—C1—C2—C3	−173.22 (13)
N1—P1—O2—C4	75.24 (15)	P1—N1—C3—O1	−0.1 (3)
O4—P1—O3—C5	−137.75 (19)	P1—N1—C3—C2	178.97 (11)
O2—P1—O3—C5	−11.5 (2)	F2—C2—C3—O1	−22.1 (2)
N1—P1—O3—C5	102.4 (2)	F3—C2—C3—O1	−140.28 (16)
F1—C1—C2—F2	68.63 (17)	C1—C2—C3—O1	97.78 (17)
F1^i^—C1—C2—F2	−175.18 (13)	F2—C2—C3—N1	158.77 (14)
C2^i^—C1—C2—F2	−53.36 (10)	F3—C2—C3—N1	40.62 (19)
F1—C1—C2—F3	−173.94 (12)	C1—C2—C3—N1	−81.33 (15)

Symmetry code: (i) −*x*+1, *y*, −*z*+1/2.

### Hydrogen-bond geometry (Å, º)

**Table:**

*D*—H···*A*	*D*—H	H···*A*	*D*···*A*	*D*—H···*A*
N1—H1*NA*···O4^ii^	0.86	1.93	2.7750 (17)	168

Symmetry code: (ii) −*x*+1, −*y*, −*z*.
